# Supplementary material for: An ABCG-Type Transporter Facilitates ABA Influx and Regulates Camptothecin Biosynthesis in Camptotheca acuminata
Source: Int J Mol Sci. 2022 Dec 17;23(24):16120. doi: 10.3390/ijms232416120 (PMC9785411; doi:10.3390/ijms232416120)
Supplement: Supplementary file 1 [file ijms-23-16120-s001.zip › ijms-2063453-supplementary/ijms-2063453-Supplementary Data/ijms-2063453-Supplementary Data.pdf]

An ABCG-Type Transporter Facilitates ABA Influx and Regulates Camptothecin Biosynthesis in Camptotheca acuminata

Yanyan Wang [1], Yang Wang [1], Hefei Bai [1], Yuqian Han [2] and Fang Yu[1] *

[1] School of Biological Engineering, Dalian Polytechnic University, Dalian 116034, China;

[2] WX Biologics, Wuxi 214000, China;

* Correspondence: yufang@dlpu.edu.cn or fyu0506@gmail.com

Table S1 Primers used in this study

Primer Name	Sequence(5'to3')
OligodT18	TTTTTTTTTTTTTTTTTT
M13F	GGTTTTCCCAGTCACGAC
M13R	AGCGGATAACAATTTCACAC
35Spromoter	CTATCCTTCGCAAGACCCTTC
NOS terminator	TGATAATCATCGCAAGACCG
TRV2Sequencing F	TGTTACTCAAGGAAGCACGATG
TRV1MP-F5600	AGATACGCCTGGGTTCATTC
TRV1MP-R5924	GGTAAAATCGCCTTCAATGT
TRV2-CP-F762	CAGTTTCCAGATAAGAAGGTGT
TRV2-CP-R1088	TTTCTCAAAGTTCCTTCGGT
CaUBC-F	CATCCAGAACCCGATAGTCC
CaUBC-R	TGTAAATTCAACCCTTTCTTGG
CaABAT-F162	TTGGTGGGTTTTCAGTTGG
CaABAT-FL-Kpn1	FGCGGTACCATGGATGGCAGTGATATTTA
CaABAT-R4671	GATCTTAGGCACTTGCTTGTG
CaABAT-FL-Sac1-Sal1-R	GCGTCGACGAGCTCTCATCGCCTTTGGAAATTGA
CaABAT-F	TTGGTGGGTTTTCAGTTGG
CaABAT-R	GAGATGCTTCTTTCTATTCGG
CaRD29B-F331	AGAGACTTGTCGAAGGAGAGTAGTG
CaRD29B-R478	CTAATTGAGTATCCTCCCTCACAGC
CaTDC1-F1543	GATTGGAAATTCCTGTGAGTG
CaTDC1-R1736	CATGAAAAGATAGGATGCGC
CaCYC1-F	TTCTGTGGTGGGTCGTGTAT
CaCYC1-R	ATTGTTGTCCAGTGCAAAGG
CaG8O-F	TCAACCTCTTCGTGCTGTTC
CaG8O-R	ATAGAACGAGAACTCCGACG
Ca7DLS-F	CCTATTTGGATCTGGGAGAC
Ca7DLS-R	TCTCTGGGCTGACATGACTAT
Ca7DLGT-F	CATCATATTGTAATTTGGACCG
Ca7DLGT-R	AACCAAATAGCTGCAGGAAT
CaSTR-F518	ACTTGAGCCAACTCAGTCAGT
CaSTR-R720	GGGACCTGTCAGCCAATA

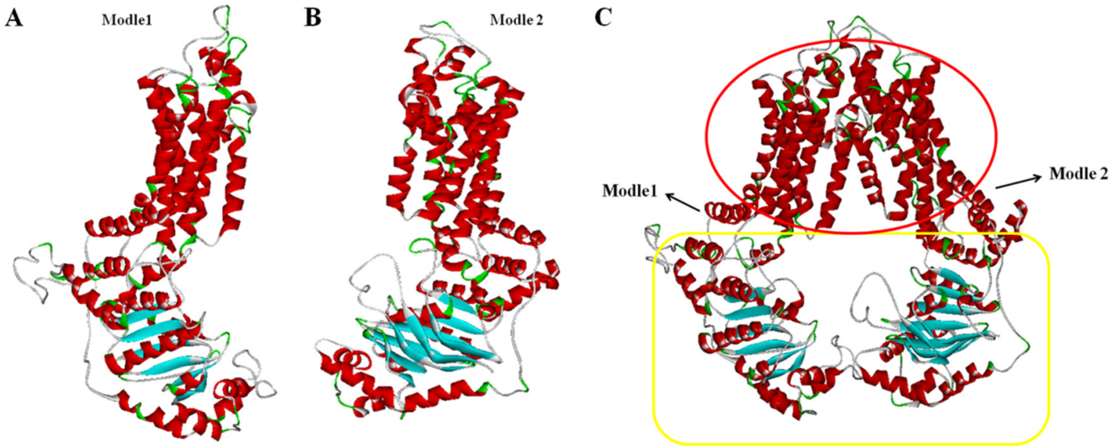

Figure S1. Homology modeling and predicted 3D structure of CaABAT using a Swiss-model server. (A), modle 1 (167-635 aa of CaABAT); (B), modle 2 (857-1455 aa of CaABAT); (C), possible 3D structure of CaABAT. (The red regions are two hydrophobic transmembrane domains, and the yellow regions are two cytosolic domains, known as the nucleotide-binding domains).

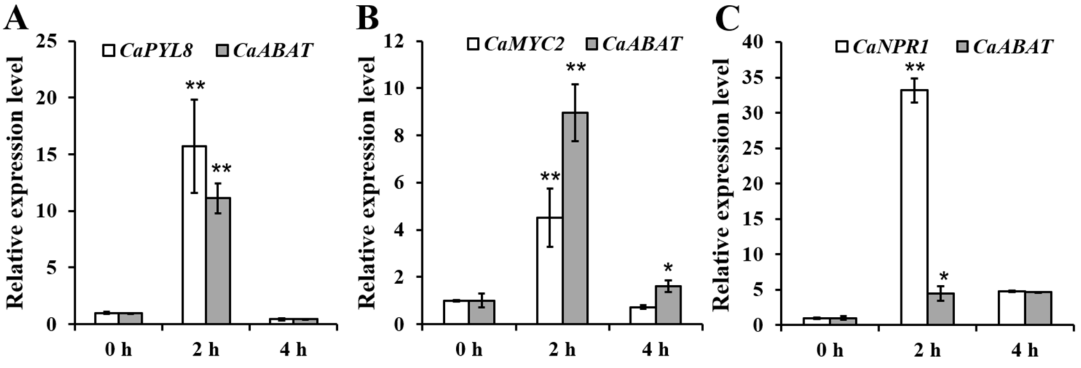

Figure S2. Relative gene expression levels in response to plant hormones treatments. (A), Relative expression levels of *CaABAT* and ABA receptor gene, *CaPYL8*, in response to 100 μM of ABA treatment. (B), Relative expression levels of *CaABAT* and JA responding gene, *CaMYC2*, in response to 20 μM of MeJA treatment. (C), Relative expression levels of *CaABAT* and SA receptor gene, *CaNPR1*, in response to 20 μM of SA treatment. The error bars represent standard deviations from three biological replicates, and asterisks indicate statistically significant differences compared with the expression levels at 0 h. *$P < 0.05$, **$P < 0.01$.

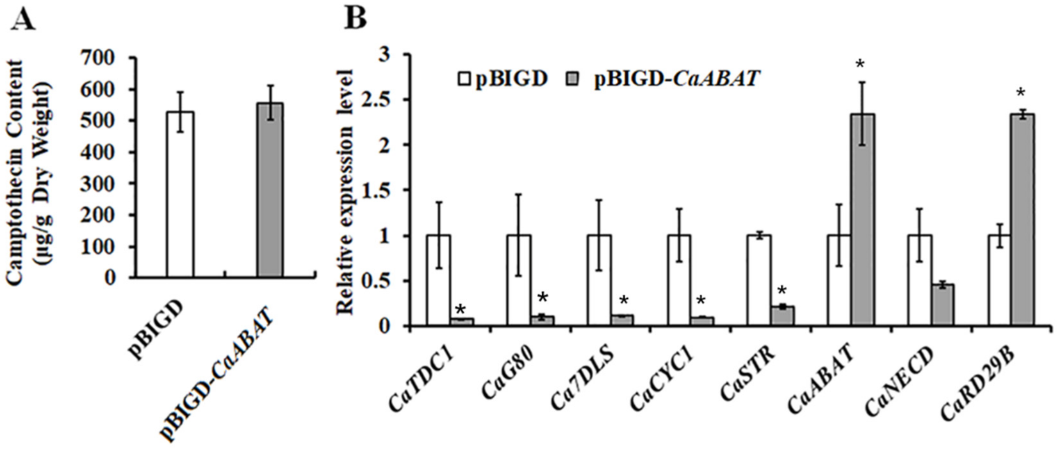

Figure S3. Overexpression of *CaABAT* in *C. acuminata* leaves. (A), CPT accumulation levels in pBIGD control and *CaABAT*-overexpressing leaves. (B), Relative expression levels of CPT biosynthesis pathway genes, ABA responding gene, *CaRD29B*, and ABA biosynthesis gene, *CaNECD* in pBIGD control and *CaABAT*-overexpressing leaves. The error bars represent standard deviations from three biological replicates, and asterisks indicate statistically significant differences compared with the expression levels in pBIGD control leaves. *$P < 0.05$.

Table S2 Motifs analysis of promoter sequences of *Ca7DLGT* and *CaG8O* with 1300 bp before transcription start site.

Name	Site Name	Sequence	Position	Matrix score	Strand	Organism	Function
ProCaG8O	ABRE	CACGTG	1036	6	-	*Arabidopsis thaliana*	cis-acting element involved in the abscisic acid responsiveness
	ABRE	ACGTG	1037	5	+	*Arabidopsis thaliana*	cis-acting element involved in the abscisic acid responsiveness
	TATC-box	TATCCCA	1239	7	+	*Oryza sativa*	cis-acting element involved in gibberellin-responsiveness
ProCa7DLGT	ABRE	ACGTG	308	5	-	*Arabidopsis thaliana*	cis-acting element involved in the abscisic acid responsiveness
	CGTCA-motif	CGTCA	127	5	+	*Hordeum vulgare*	cis-acting regulatory element involved in the MeJA-responsiveness
	CGTCA-motif	CGTCA	342	5	-	*Hordeum vulgare*	cis-acting regulatory element involved in the MeJA-responsiveness
	TGACG-motif	TGACG	127	5	-	*Hordeum vulgare*	cis-acting regulatory element involved in the MeJA-responsiveness
	TGACG-motif	TGACG	342	5	+	*Hordeum vulgare*	cis-acting regulatory element involved in the MeJA-responsiveness
