# Supplementary figures and images for: An ABCG-Type Transporter Facilitates ABA Influx and Regulates Camptothecin Biosynthesis in Camptotheca acuminata
Source: Int J Mol Sci. 2022 Dec 17;23(24):16120. doi: 10.3390/ijms232416120 (PMC9785411; doi:10.3390/ijms232416120)

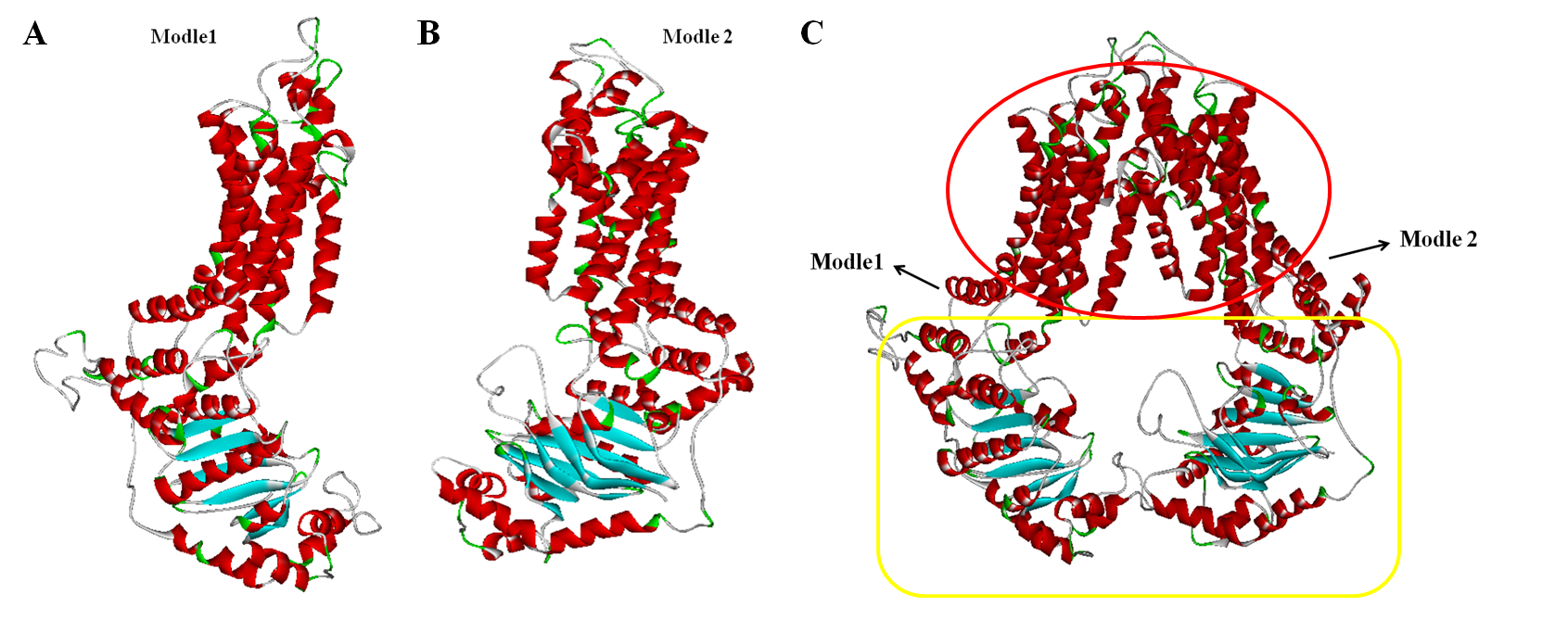

Supplement: Supplementary file 1 [file ijms-23-16120-s001.zip › ijms-2063453-supplementary/ijms-2063453-Supplementary Data/Figure.S1.tif]

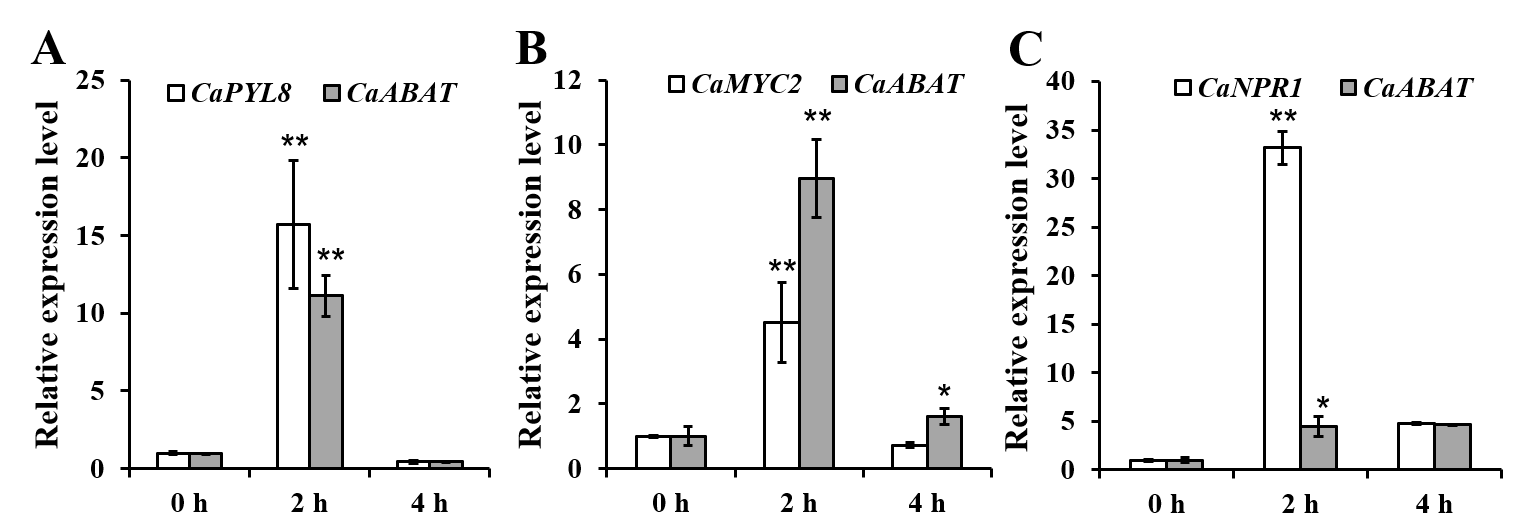

Supplement: Supplementary file 1 [file ijms-23-16120-s001.zip › ijms-2063453-supplementary/ijms-2063453-Supplementary Data/Figure.S2.tif]

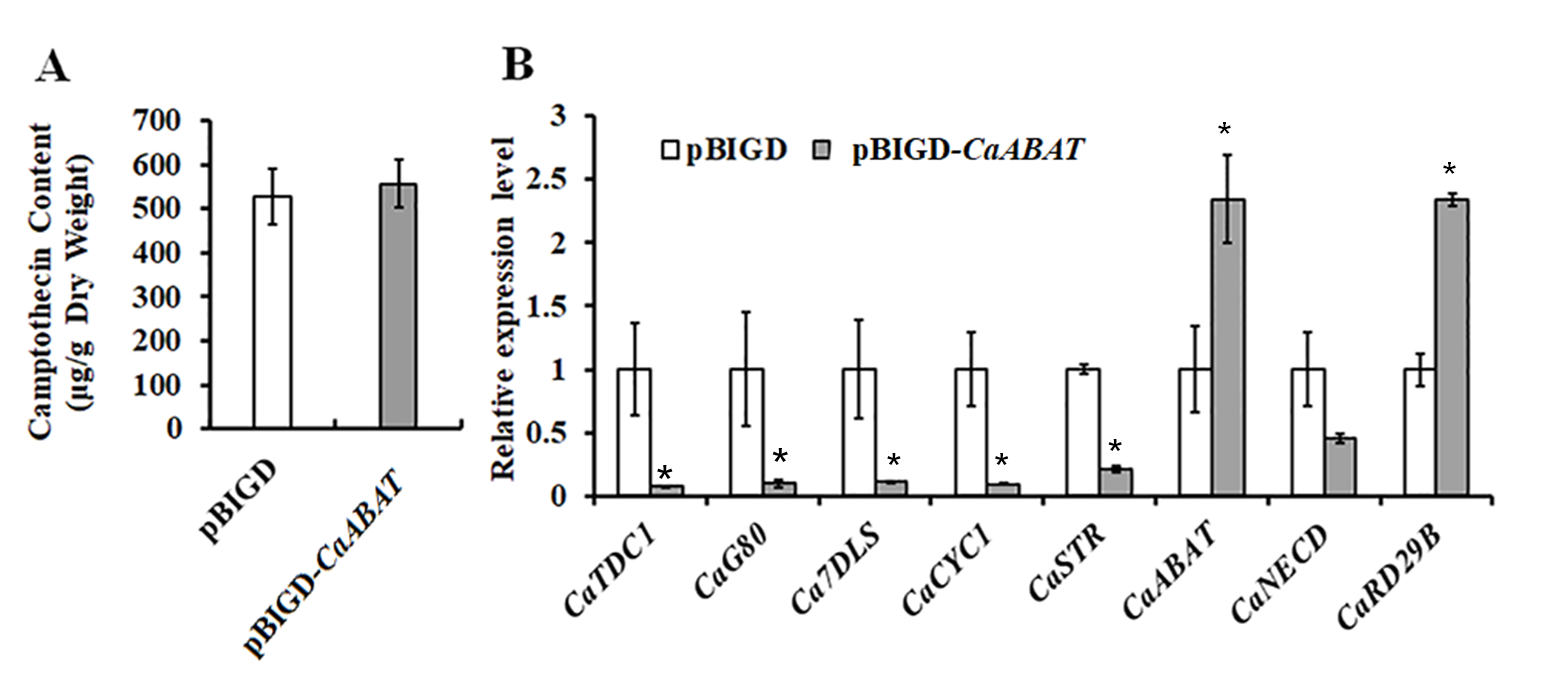

Supplement: Supplementary file 1 [file ijms-23-16120-s001.zip › ijms-2063453-supplementary/ijms-2063453-Supplementary Data/Figure.S3.tif]
